# The Impact of Visual Impairment on Completion of Cognitive Screening Assessments: A Post-Hoc Analysis from the IVIS Study

**DOI:** 10.22599/bioj.263

**Published:** 2022-06-30

**Authors:** James Bould, Lauren Hepworth, Claire Howard, Jim Currie, Fiona Rowe

**Affiliations:** 1 University of Liverpool, GB; 2 Northern Care Alliance NHS Foundation Trust, GB; 3 VISable, GB

**Keywords:** Visual symptoms, Visual impairment, Stroke, Vision, Refractive Error, Cognitive, Cognition, OCS

## Abstract

**Aim:**

The aim of this study was to evaluate completed cognitive screens in stroke survivors with and without visual impairment to explore whether the presence of visual impairment impacts on completion of cognitive screening.

**Materials and methods:**

Cognitive screening assessment was undertaken using the Oxford Cognitive Screen (OCS). Data from visual function assessments (inclusive of visual acuity, visual fields, eye movements and visual perception evaluation) were analysed to determine whether presence and/or type of visual impairment impacted on cognitive screening scores achieved. Covariates, including glasses use, gender, age at stroke onset and stroke type, were used to assess confounding impacts on scores attained during cognitive screening.

**Results:**

1500 stroke admissions were recruited. One hundred ninety-seven who completed the OCS, were identified from the IVIS study database. Those who reported visual symptoms performed worse statistically on all cognitive tasks except the recall recognition (p = 0.232) and executive tasks (p = 0.967). Visual symptoms did not prevent participants from completing every section of the OCS (p = 0.095). In certain tasks, those not wearing their required glasses performed worse, including the executive function (p = 0.012), broken hearts and sentence reading tasks.

**Conclusions:**

Many tasks within cognitive screening assessment are impacted by presence of visual deficits, and adjustments, where possible (e.g. good lighting, large print) should be used to facilitate completion of cognitive screening. It is important to ensure required reading correction is worn during screening.

## Introduction

The World Health Organization defines stroke as ‘rapidly developing clinical signs of focal or global disturbance of cerebral function, with symptoms lasting 24 hours or longer or leading to death, with no apparent cause other than of vascular origin’ (World Health Organization 2020). It is the second most common cause of death worldwide, with the number of stroke-related deaths between 1990–2010 increasing by 26% (Hankey 2014). However, in the UK, stroke incidence has decreased and post-stroke survival has improved over the last decade (Kalaria et al. 2016). Nonetheless it remains the primary cause of interminable and acquired disability (Sarikaya et al. 2015). Approximately 80% of stroke survivors encounter acute cognitive impairment, persisting long-term in 38-73% of cases (Patel et al. 2003; Leśniak et al. 2008). A key aetiology is vascular dementia, which is thought to develop in approximately 38% of stroke survivors and can significantly affect cognitive ability and activities of daily living (ADL) (Kalaria et al. 2016). It is also approximated that 60% of stroke survivors experience visual impairments; including visual field loss, visual inattention, ocular motility defects, visual perception issues and reduced visual acuity (Rowe et al. 2019).

The Oxford Cognitive Screen (OCS) has been specifically developed for cognitive assessment in stroke populations (Demeyere et al. 2015). The OCS comprises ten tasks used to assess five cognitive domains: attention and executive function, memory, language, number processing and praxis, and takes approximately 15 minutes to complete (Demeyere et al. 2016). This tool allows rapid assessment of cognitive function, thereby acting as an indicator as to whether further neurological assessment is necessary, should deficiencies in any of the cognitive domains be discovered. The OCS was devised to be inclusive of patients with aphasia, visuo-spatial inattention (neglect), visual field loss, apraxia and reading/writing problems (Demeyere et al. 2015; Demeyere et al. 2016). This was achieved by utilising vertical layouts, multimodal presentations, forced-choice examination techniques and short high-frequency words (Demeyere et al. 2015). Moreover, tasks were devised to discover multiple impairments simultaneously to make the screen time-efficient, for example the sentence reading task also assesses memory. Despite the OCS having been developed for stroke populations, reports of its use in visually impaired populations post-stroke are lacking. The aim of this study was to evaluate completed cognitive screening assessment in stroke survivors with and without visual impairment to explore whether the presence of visual impairment impacted on completion of OCS cognitive screening.

## Materials and Methods

### Recruitment

The IVIS (Impact of Visual Impairment after Stroke) study has been reported previously (Rowe et al. 2019). In summary, the study was conducted in three acute stroke units across North-West England. All stroke admissions were recruited over 15 months (1^st^ July 2014 – 30^th^ September 2015) in a prospective epidemiological study. Ethical approval was attained from the Health Regulatory Authority (Research Ethics Committee reference 14/NW/0166) and the study was undertaken in accordance with the Tenets of Helsinki. The inclusion criteria were for age over 18 years and confirmed diagnosis of stroke.

### Assessment

Visual assessments were undertaken by stroke specialist orthoptists which comprised of a clinical history including visual symptoms and authenticated clinical measurements of visual acuity, reading ability, colour vision, ocular alignment test, lid and pupil function, rotation of eye movements, vergence, stereopsis, fusional vergence, visual field assessment, visual perception and visual inattention (Rowe et al. 2019). Visual symptoms were defined as patient-reported subjective accounts of what they notice in regard to their visual abilities and visual impairments are defined as visual conditions objectively identified from formal visual assessment. Management for visual impairment would have been given at the time of this assessment if appropriate, e.g. occlusion or prism for diplopia. Stroke severity was scored using the Barthel scoring system which is a measure of independence in ADLs (Ohura et al. 2017). Cognitive screening was undertaken by a member of multi-disciplinary team during the inpatient stay, if possible. This was a pragmatic study and the order of the vision assessment and cognitive screen was dependent on availability of the orthoptist or multi-disciplinary team for these assessments. The paper-based OCS was one of the tools used to assess cognition; domains and task description along with score ranges and the indication of an impaired score are outlined in Table 1 (Demeyere et al. 2015). These assessments were attempted as soon as possible during the inpatient stay. There was some overlap in the assessments of the OCS and the visual assessment; primarily assessments of visual field and visual inattention. Both assessments were conducted using the corresponding instructions/technique.

**Table 1:** Domains and cognitive tasks for Oxford Cognitive Screen (OCS), score range and impaired score indication (Demeyere et al. 2015).


DOMAIN	TASK DESCRIPTION (SCORE RANGE)	IMPAIRED SCORE

Language	Picture naming (0–4)	<3

	Semantics (0–4)	<3

	Sentence reading (0–4)	<4

Memory	Orientation (0–4)	<4

	Recall and recognition – verbal and episodic memory (0–4)	<3

Number Processing	Number writing (0–3)	<3

	Calculation (0–4)	<3

Visual Attention and Executive Function	Broken hearts cancellation (–25–25)	>1 Left neglect <–1 Right neglect

	Trail making (–13–12)	>0

Praxis	Imitation of meaningless gestures (0–12)	<8

Visual Fields	Confrontation (0–4)	<4


### Patient involvement

This study addresses a key research priority identified by patients and the public: “What is the most effective way to assess vision with neurological visual impairment i.e., stroke, dementia and cerebral/cortical visual impairment?” (Rowe et al. 2014). Consultations with patients from the UK VISable stroke and vision patient, carer and public involvement (PCPI) panel occurred whilst designing the study and for study monitoring purposes reports were disseminated to the panel.

### Analysis

Data regarding stroke type and laterality, brain region affected, age at stroke, gender, ethnicity and stroke-severity were collected and assessed for differences in OCS scores achieved. Percentages of stroke survivors for whom the OCS was not administered, alongside those who were unable to complete specific domains of either screen, were examined to give descriptive results. Chi-square tests were used to evaluate distributions in gender and stroke type. Similarly, Kruskal-Wallis tests were completed for age and duration between stroke and full visual assessment. Fisher’s Exact test for categorical data was used to assess if visual symptoms impacted on scores. Additionally, one-way ANCOVA tests were used to evaluate if gender or age affected OCS scores achieved for each screen. Independent samples T-tests were used to assess if wearing glasses impacted on scores. Mann-Whitney-U tests were then utilised to provide visualisation of any statistical significance between OCS scores when compared to having visual symptoms or not. Finally, one-way ANOVA analyses were conducted to assess whether diagnosed visual deficits impacted on OCS scores. All statistical analyses were conducted using IBM SPSS Statistics, version 25 (IBM Corp., Armonk, N.Y., USA).

## Results

**Figure 1:**
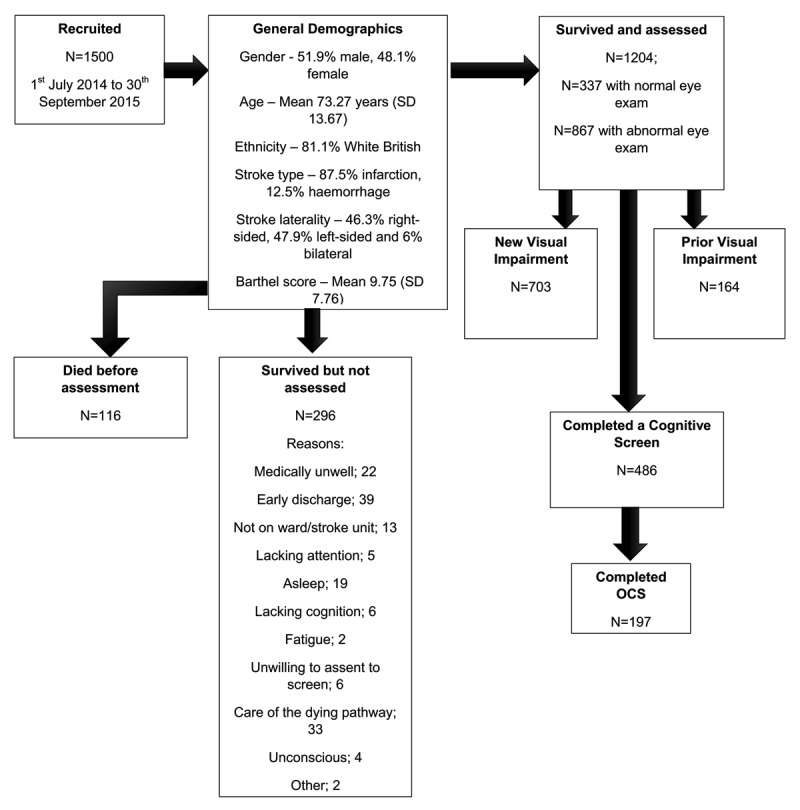
Flow diagram of participants through epidemiology study to OCS completion.

One thousand five hundred stroke admissions were recruited from three acute stroke units, as part of an epidemiology study; these results are presented elsewhere (Rowe et al. 2019). This analysis presents data from a sub-group that were able to undertake vision and cognitive assessments. Participant flow through the study is outlined in Figure 1.

**Figure 2:**
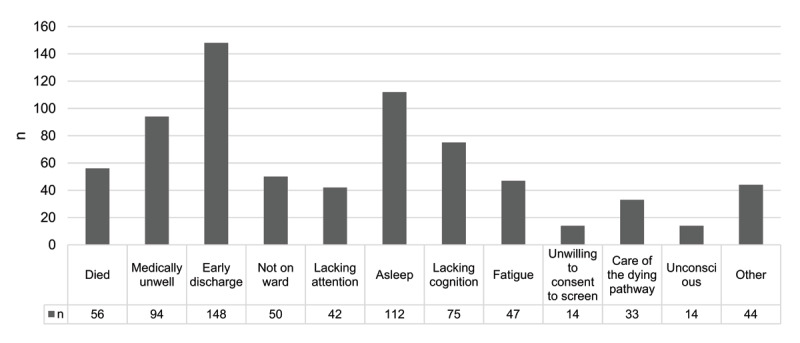
Number of stroke survivors where cognitive screening was not administered.

Of the 1204 stroke survivors that had visual assessments, 486 (40.4%) undertook cognitive screening using a range of different tools (475 within the first week post-stroke, 11 in the second week). Figure 2 highlights reasons for 729 non-assessments within the first week; repeated attempts to complete screening were made. One hundred and ninety-seven individuals underwent the OCS (40.5%).

Of the 197 individuals that undertook the OCS, 101 were female and 96 were male, consisting of white British (n = 191), white Irish (n = 1), white other (n = 4) and Bangladeshi (n = 1) individuals. The mean age at stroke was 71.2 years (SD 13.8). Type of stroke was infarction in 91.9% and haemorrhagic in 8.1%, right-sided in 49.7%, left-sided in 46.2% and bilateral in 4.1%. Overall OCS score evaluation was completed for each subsection to allow for later comparisons. The possible range of scores and the cut off for an impaired score are outlined in Table 1. Cognitive impairment was indicated in at least one domain for 174 (88.3%) stroke survivors.

Of the 197 stroke survivors that attempted the OCS, 13 (6.6%) were unable to complete the full screen. Of those unable to complete the OCS, 84.6% were diagnosed with at least one visual impairment (Table 2). The tasks which were not completed are outlined in Table 3.

**Table 2:** Completion rates of the Oxford Cognitive Screen (OCS) with and without visual impairment, n = 197.


	OCS WITH VISUAL IMPAIRMENT n = 147	OCS WITH NORMAL VISUAL FUNCTION n = 47	OCS WITHOUT VISUAL ASSESSMENT n = 3

OCS fully completed	136 (73.9%)	45 (24.5%)	3 (1.6%)

OCS partially completed	11 (84.6%)	2 (15.4%)	0


**Table 3:** Number and percentage of stroke survivors where tasks of the Oxford Cognitive Screen (OCS) were not administered.


DOMAIN	TASKS	n	%

Language	Picture naming	0	0

	Semantics	1	0.5

	Sentence reading	3	1.5

Memory	Orientation	1	0.5

	Recall and recognition (verbal and episodic)	3	1.5

Number	Number writing and calculation	3	1.5

Visual perception and executive function	Broken hearts	8	4.1

	Trail making	10	5.1

Praxis	Gesture	4	2.0

Visual field	Visual fields	2	1.0


*‘n’ represents the 13 individuals that were unable to complete the full OCS*.

These results highlight that the tasks of the ‘visual perception and executive function’ domain were among those completed least often. Non-completion was due to a mix of presence of visual impairment impeding completion as well as presence of cognitive impairment; the main reasons identified were an inability to see the broken hearts (i.e. presence of visual defect), declining to complete this section of the screen (typically due to fatigue), and generally being unable to complete these tasks (i.e. presence of cognitive impairment or not fully understanding the concept of the task).

Table 4 provides information detailing average scores on the various OCS tasks according to gender, in addition to distinctive age brackets. After adjusting for age, there were no significant statistical differences between genders for any of the OCS tasks (p > 0.05). However, after adjusting for gender, there were statistically significant differences between ages (p < 0.05) for four tasks: picture naming (p = 0.008), recall/recognition (p = 0.044) numbers (p = 0.039) and the executive task (p = 0.041). Younger age groups performed better on each of these tasks, with the exception of the executive task where the 65–75 years age group performed better.

**Table 4:** Average scores achieved for both genders and across different age ranges.


TASK NAME (OPTIMUM SCORE)	MEASURE	OVERALL	MALE (n = 96)	FEMALE (n = 101)	p	<65 (n = 59)	65–75 (n = 52)	>75 (n = 86)	p

**Picture naming** **(4)**	Overall accuracy	3.16	3.23	3.10	0.778	3.47	3.33	2.85	0.008*

**Semantics** **(4)**	Overall accuracy	2.80	2.84	2.76	0.564	2.88	2.86	2.71	0.264

**Sentence reading** **(15)**	Overall accuracy	12.34	12.56	12.13	0.569	11.83	13.00	12.14	0.516

**Orientation** **(4)**	Overall accuracy	3.63	3.67	3.59	0.598	3.71	3.75	3.51	0.207

**Recall and recognition** **(8)**	Verbal memory and episodic memory	6.29	6.78	5.83	0.097	6.71	6.94	5.54	0.044*

**Numbers (writing and calculation)** **(7)**	Overall accuracy	5.42	5.73	5.13	0.064	5.72	5.67	5.00	0.039*

**Broken hearts** **(0)**	Overall accuracy Asymmetry (left egocentric neglect > 0, right < 0)	1.35	1.88	0.83	0.348	0.52	3.10	0.87	0.211

**Executive task** **(**–**1)**	Overall accuracy	1.69	1.43	1.95	0.566	1.23	0.78	2.56	0.041*

**Praxis/imitation** **(12)**	Overall accuracy	10.98	11.05	10.91	0.918	11.47	11.21	10.50	0.097

**Visual fields** **(4)**	Overall accuracy	3.73	3.75	3.72	0.956	3.81	3.73	3.68	0.687


* Significance; <0.05.

### OCS completion and visual symptoms

Of the 486 stroke survivors who attempted the OCS, 296 (60.9%) reported no visual symptoms, with 173 (35.6%) reporting visual symptoms and 17 (3.5%) unable to report the presence or absence of visual symptoms. The proportions of stroke survivors experiencing different types of visual symptoms post-stroke are reported in Table 5. The differences in scores achieved for each subsection of the OCS for those with and without visual symptoms was assessed. The possible range of scores and the cut off for an impaired score are outlined in Table 1. Almost all tasks revealed statistically significant results (p < 0.05), with those reporting symptoms performing worse, except recall recognition (p = 0.232) and executive tasks (p = 0.967).

**Table 5:** Ability to complete Oxford Cognitive Screen (OCS) with and without visual symptoms.


VISUAL SYMPTOMS	N (%)	FULL COMPLETION OF OCS	PARTIAL COMPLETION OF OCS	TOTAL ATTEMPTING OCS

No symptoms	296 (60.9)	116	8	124

Visual symptoms	173 (35.6)	63	3	66

Reading difficulty	17 (3.5)	4	0	4

Blurred, altered or reduced vision	64 (13.2)	28	2	30

Field loss	28 (5.8)	11	1	12

Diplopia	24 (4.9)	9	0	9

Other	40 (8.2)	11	0	11

Not able to report	17 (3.5)	6	1	7

Total		185	12	197


Other symptoms included oscillopsia, visual hallucinations, colour problems, image movement problems, visual illusions, visual disorientation, dry/gritty eyes, eye strain, watering eyes, photophobia, inattention.

Table 5 outlines the number of stroke survivors with and without visual symptoms that fully or partially completed the OCS. Of the 197 individuals that undertook the OCS, 184 (93.9%) fully completed the screen with partial completion by 13 (6.6%). Of these 184 stroke survivors completing the OCS 34.2% reported visual symptoms. Of the 13 stroke survivors that partially completed the OCS, 23.1% reported visual symptoms and 15.4% were unable to report if they had any symptoms or not. Notably similar proportions of stroke survivors fully completed the OCS with no visual symptoms (93.5%) and with visual symptoms (95.5%) (p = 0.095).

### Glasses not available

**Figure 3:**
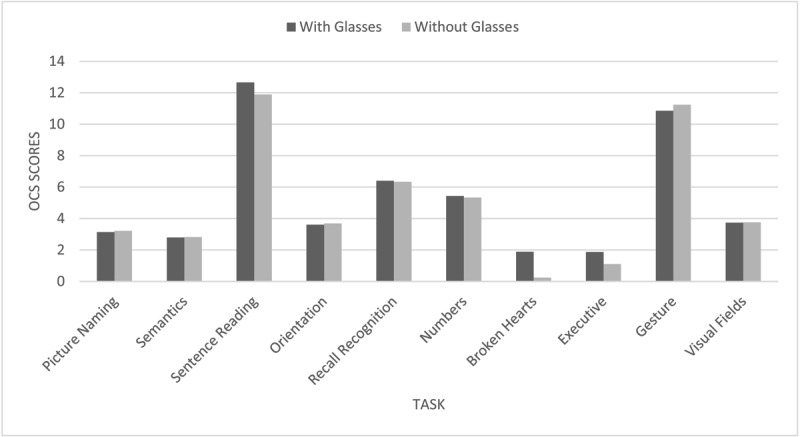
OCS mean scores achieved with and without glasses. Each task has a different scoring range and therefore should not be compared to each other, the purpose of this figure is to allow comparison of each task completed by individuals with or without glasses.

Glasses were normally worn by 186 (94.4%), 9 (4.6%) did not wear glasses and for 2 (1.0%) stroke survivors, it was unknown whether they regularly wore glasses or not. However, 38 individuals (19.3%) who ordinarily wore glasses, did not have them available to wear them during the OCS. Analysis was undertaken to assess if the results of those who wore their glasses during the screen performed better than those who normally wore glasses but did not have them available to wear them during the screen. Table 6 highlights these results, displaying the number of stroke survivors scoring the optimum for each task and Figure 3 displays the comparison of the mean scores. The optimum score refers to the best score which does not meet the criteria of cognitive impairment (outlined in Table 1). This does not equate to the maximum score for all tasks i.e. broken hearts and executive function tasks.

**Table 6:** OCS optimum scores achieved (best score which does not meet the criteria of cognitive impairment) with (n = 147) and without (n = 38) glasses. OCS scores for participants wearing glasses compared to those who normally wear glasses but did not during the screen. Also inclusive of mean scores achieved. * <0.05.


TASK NAME (OPTIMUM SCORE)	n (WITH GLASSES)	%	MEAN	n (WITHOUT GLASSES)	%	MEAN	p

**Picture naming (4)**	84	57.1	3.14	23	60.5	3.21	0.817

**Semantics (3)**	129	88.4	2.79	33	86.8	2.82	0.653

**Sentence reading (15)**	101	70.1	12.65	22	57.9	11.89	0.316

**Orientation (4)**	112	76.7	3.61	32	84.2	3.68	0.541

**Recall recognition (12)**	11	7.6	6.40	1	2.6	6.34	0.122

**Numbers (7)**	58	40.3	5.44	13	34.2	5.34	0.983

**Broken hearts (0)**	37	26.4	1.88	10	26.3	0.24	0.072

**Executive task (–1)**	6	4.3	1.87	0	0	1.11	0.012*

**Gesture (12)**	115	80.4	10.85	31	81.6	11.24	0.133

**Visual fields (4)**	129	89	3.74	33	86.8	3.76	0.118


Influence of visual function deficits.

Sentence reading had a 12.2% difference in those achieving the optimum score between those with and without glasses. For the executive task, of those without glasses none achieved the optimum score, with the highest score achieved being 10 and found to be statistically significant difference (p = 0.012).

Scores of the OCS were evaluated to assess whether there any difference between those with a normal vision assessment and visual impairment, encompassing abnormalities in visual acuity, ocular alignment, ocular movement, binocular vision, visual fields, visual neglect and visual perception. Ocular alignment, ocular movement and binocular vision were grouped into the category ‘abnormal eye movement,’ as they are interlinked and it was very likely participants had more than one of these visual function deficits. Participants with more than one visual impairment diagnosed, such as visual field loss and visual inattention, were part of the analysis for each relevant type of visual impairment. Table 7 outlines the numbers of individuals that undertook the cognitive screen experiencing one or more of these visual defects. Stroke survivors with and without each of the types of visual impairment were compared in their performance of each task on the OCS. The p values in Table 7 indicate the significance of the difference for this comparison.

**Table 7:** Visual impairment diagnosis of those that undertook each cognitive screen with p values broken down by task. * <0.05, ** <0.001.


n = 197	VISUAL ACUITY	EYE MOVEMENT	VISUAL FIELD	VISUAL INATTENTION	VISUAL PERCEPTION

**Participants n (%)**	116 (58.9)	83 (42.1)	46 (23.4)	33 (16.8)	7 (3.6)

**Picture naming**	0.000**	0.000**	0.000**	0.000**	0.757

**Semantics**	0.000**	0.001*	0.000**	0.000**	0.000**

**Sentence reading**	0.000**	0.010*	0.000**	0.000**	0.163

**Orientation**	0.000**	0.047*	0.005*	0.000**	0.838

**Recall recognition**	0.725	0.450	0.302	0.691	0.074

**Numbers**	0.015*	0.091	0.009*	0.840	0.751

**Broken hearts**	0.265	0.019*	0.347	0.112	0.913

**Executive**	0.688	0.190	0.150	0.378	0.632

**Gesture**	0.000**	0.001*	0.000**	0.000**	0.004*

**Visual fields**	0.000**	0.003*	0.000**	0.002*	0.000**


A significantly worse performance was detected on the following tasks in the OCS in the presence of visual acuity and visual field abnormalities; picture naming, semantics, sentence reading, orientation, numbers, gesture and visual field. Similarly, the presence of visual neglect was associated with a worse performance in the picture naming, semantics, sentence reading, orientation, gesture and visual field tasks. The presence of an eye movement disorder was associated with statistically significant poorer performance in the picture naming, semantics, sentence reading, orientation, gesture, visual fields and broken hearts tasks. Finally, those with visual perception abnormalities had statistically significant poorer performance in three tasks; semantics, gesture and visual fields.

Comparatively, a diagnosis of reduced visual acuity, a visual field defect or visual inattention had a statistically significantly poorer performance on the picture naming and orientation tasks. Visual perception was associated with statistically significant poorer results in three tasks; picture naming, language and abstraction tasks.

## Discussion

The Montreal Cognitive Assessment (MoCA) and the Mini Mental State Examination (MMSE) are well documented and widely used methods for screening individuals for cognitive impairments (Pendlebury et al. 2010; Ciesielska et al. 2016). However, neither were designed specifically to assess cognitive function for stroke populations but rather to evaluate individuals with dementia and give a general cognition score with a single cut-off score (Mancuso et al. 2018). The OCS was the first stroke-specific cognitive screening tool which addressed issues identified with the MoCA and MMSE for a stroke population, and therefore is considered to be more sensitive in this population (Demeyere et al. 2016; Mancuso et al. 2016). Our study evaluated the use of the OCS in a population assessed specifically for visual impairments to determine whether presence of visual impairment impacted on the completion of the OCS screening assessment.

The OCS results revealed a high incidence of cognitive impairment (88.3% in at least one cognitive domain), which was higher than figures of cognitive impairment reported in other studies at the acute stage post-stroke of 72% to 80% (Nys et al. 2007; Leśniak et al. 2008; Renjen et al. 2015). This could indicate that cognitive impairment is being overestimated in the presence of visual impairment. Both visual impairment and cognitive impairment are both highly at risk of underestimation without screening (Chan et al. 2014; Hepworth et al. 2021).

Covariates including age, visual symptoms and diagnosed visual impairments revealed statistically significant differences, whereas gender did not. Poorer OCS scores were found with certain tasks (sentence reading and numbers tasks) as age increased. Increasing age has been reported to be associated with increased incidence of stroke and dementia, both of which can impact cognitive function (Lo Coco et al. 2016). However, visual impairment and requirement for refractive correction is also more prevalent in older age (Lotery et al. 2000; Evans & Rowlands 2004). The association in our study for poorer OCS scores and increasing age likely reflect a combination of cognition and visual impairments impeding task completion. Results also revealed that individuals reporting visual symptoms performed statistically worse on all tasks of the OCS except the executive function and recall tasks. Visual symptoms therefore represent a potentially confounding factor. Similarly, it was identified that stroke survivors with various diagnosed visual impairment scored significantly worse in multiple tasks of the OCS (notably the semantics, gestures and visual fields tasks). These results highlight the importance that vision impairments have upon the stroke survivors’ abilities to complete the various tasks of the OCS and that, commonly, a poorer score may not necessarily be attributed to cognitive ability, but to the presence of visual impairment.

Glasses use during the OCS was another potential confounding factor that was explored as 38 (19.3%) individuals who normally wore glasses did not have them available to wear them during the screen. Each task was analysed independently, with key tasks based on visual acuity (i.e. clarity of central vision) requirement including picture naming, semantics, sentence reading, broken hearts, executive task, gesture and visual fields. Ability to complete, and level of indicated cognition in 9 out of 10 tasks were similar between the two groups, with the exception of the executive task. This highlights the importance of ensuring all participants wear their glasses to prevent inaccurate indications of cognitive ability being attained. Furthermore, it is important to consider the small population that this analysis was conducted on. Whilst not statistically significant in this small sample, other tasks such as broken hearts may be impacted by the correct refractive prescription being unavailable. Ensuring stroke survivors have their glasses available in hospital is imperative for such assessments. It is possible a larger cohort could reveal a higher impact and research is warranted to evaluate this further. The unavailability of glasses whilst an inpatient in hospital is a common problem. Lotery et al. (2000) reported 25% of stroke survivors did not have their glasses available in hospital and 27% had dirty or damaged glasses. Visual impairment resulting from lack of accessibility to refractive correction is correctable and would easily improve the accuracy of cognitive screening (Lotery et al. 2000).

Both the vision and cognitive assessment were conducted in the acute stage post-stroke. Studies have shown that is it possible, acceptable and accurate to conduct screening at this early time point (Demeyere et al. 2014; Rowe et al. 2019). Early identification of both vision and cognitive deficits allows for adaptation of rehabilitation to increase the possibility of stroke survivors reaching their rehabilitation potential (Whitson et al. 2017). Our study highlights that visual impairment is common in stroke survivors in the acute stage and that this must be accounted for when clinicians are conducting assessments that involves near tasks whether that is cognitive screening or other types of assessment. Knowing about presence of visual impairment is therefore very important to improve the reliability of screening.

### Limitations

Due to the small sample and post-hoc methodology of this analysis, caution should be exercised in the interpretation of results. For example, 38 (19.3%) of the 197 OCS participants did not have glasses available to wear during the screen that they would normally be worn. This small percentage could be misrepresentative and further research is required to explore the impact of not having correct refractive reading correction available when completing assessments such as the OCS. The need to wear glasses is obvious when attention is drawn to this issue. However, this was a pragmatic study and demonstrates that even this basic visual need was not attended to on the stroke unit. Many stroke survivors could not be screened due to factors such as fatigue, being medically unwell or lacked adequate cognition to consent to or comply with cognitive screening. It is commonly recognised that more severe and extensive strokes are likely to cause amplified cognitive impairments (Makin et al. 2013). It is likely that a number of these individuals had impaired cognitive ability of some degree, or visual impairments that were too severe to allow assessment to be conducted (Rowe et al. 2019). There may have been a variation in the time between the OCS and the visual assessment being conducted. It is possible for spontaneous improvement in the visual deficit and/or cognitive status to have occurred between the two assessments. In future studies the time between assessments should be minimised.

## Conclusions

Results of this study indicate that despite the OCS being reported as highly sensitive for cognitive impairments post-stroke in previous studies, visual problems can significantly impact upon OCS assessment and outcome. It is important to detect these pre-existent or incidental visual changes, to inform the assessment and subsequent consideration of this when interpreting the scoring. In addition, it may be beneficial for an adaptation of the paper-based OCS to be made that mitigates against the impact from visual impairment. Improved accuracy of visual and cognitive screening is likely to provide benefits to carers, staff and patients as early identification can lead to better and more personalised care. Such adaptation will require further research and validation.
